# Contradictory findings in the study of emotional false memory: a review on the inadvisability of controlling valence and arousal

**DOI:** 10.3389/fpsyg.2024.1380742

**Published:** 2024-05-28

**Authors:** Haochen Yin, Yizhou Zhou, Zuoshan Li

**Affiliations:** ^1^Key Laboratory of Applied Psychology, Chongqing Normal University, Chongqing, China; ^2^School of Teacher Education, Chongqing Normal University, Chongqing, China

**Keywords:** false memory, emotion, mood, emotional valence, emotional arousal

## Abstract

Emotional false memories are the erroneous recollection of events accompanied by an emotional experience. In high-risk domains like psychotherapy and the legal system, emotional false memories are of particular importance. Despite the systematic research conducted on emotional false memories in recent years, findings remain contradictory. Some studies have suggested that negative emotion reduces false memories, while others have suggested that negative emotion increases false memories. Research has mainly employed words and pictures as experimental stimuli, and studies using both types of memory stimuli are reviewed here. From this examination, it emerged that the main reasons for contradictory findings are as follows: (1) different materials have varying effects on inducing false memories, with pictures demonstrating a memory advantage compared to words; (2) recall and recognition tests have been used interchangeably, leading to different false-memory effects depending on the memory test employed; and (3) different studies have adopted different levels of control over valence and arousal when manipulating emotional variables. Future studies should distinguish between the use of different memory materials, examine specific differences in recall and recognition tests, and measure the impact of specific emotions on false memory beyond the dimensions of valence and arousal.

## 1 Introduction

Memory is not a faithful reproduction of an individual’s experiences but a reconstruction process that often leads to errors. These errors can be harmless, such as when people misremember what they had for dinner, but they can also have serious consequences. In the field of law, for example, when there is a lack of direct physical evidence relating to a crime, the evidence used to accuse and convict the defendant largely relies on memory (Brainerd and Reyna, 2019). In the field of psychotherapy, therapeutic techniques such as hypnosis and dream interpretation can increase the likelihood of inducing harmful false memories, with an average of 20–50% of individuals experiencing the induction of false events (Muschalla and Schönborn, 2021). In the medical field, patients reporting their symptoms incorrectly or doctors misremembering a condition can lead to misdiagnosis and subsequent adverse effects on health. In the existing literature, false memories can be classified into two categories (Ost et al., 2013): (1) implanted false memories induced by suggestion and (2) spontaneous false memories generated without any external pressure. Among them, the former is an external distortion, while the latter is an internal distortion. These two types of false memories are only weakly correlated with one another (Calvillo and Parong, 2016; Bernstein et al., 2018), and this article focuses on the latter.

Regarding the theory of emotions, the majority of studies probing the impact of emotions on false memory are grounded in the circumplex model of emotion (Russell, 1980), which posits that emotional experiences are composites constructed from various dimensions of valence and arousal—for instance, positive, high-intensity emotional experiences signifies happiness; positive, moderate-intensity emotional experiences denotes relaxation; negative, moderate-intensity emotional experiences signify tedium; and negative, extreme-intensity emotional experiences symbolize fear. The dominant position of the circumplex model of emotion within this sphere can largely be attributed to the availability of a standardized tools, such as the International Affective Picture Series (IAPS) (Lang et al., 1998), while available standardized materials grant researchers the capacity to manipulate the valance and arousal of the material. In recent years, empirical research has provided a wealth of evidence on the impact of emotional stimuli on false memory (Kensinger and Schacter, 2008), and the debate about when and how emotions affect false memory has continued. Regarding the theory on the influence of emotions on false memories, some theories explain the impact of emotions on false memories from different perspectives. One theory, Emotional Enhancement of Memory (Heuer and Reisberg, 1990), explains the impact of emotions on memory from a broad perspective, suggesting that emotional content can enhance the accuracy of memory; for example, people tend to remember events that are accompanied by intense emotions. In contrast, the Distinctiveness Heuristic Account makes more specific predictions, suggesting that negative emotions are less likely to generate false memories; this theory posits that people remember the distinctive details of events they have experienced and make recognition judgments based on these distinctive details (Schacter and Wiseman, 2006)—for instance, “I clearly recall that the dragon fruit I consumed last week had white flesh, not red flesh, as red-fleshed dragon fruits are quite distinctive.” Emotional content is often more distinctive and can serve as a cue for a distinctiveness heuristic (Schacter et al., 2011); specifically, negative emotional content is highly relevant to survival, and individuals are more likely to accurately remember negative emotional content, making it less prone to producing false memories. These two theories offer different predictions about the specific direction of the impact of negative emotions on false memory, which is core to the ongoing debate. The concept of emotional memory trade-off effects (Kensinger et al., 2007) outlines a contrast in how emotional and neutral components of an event are remembered; while emotional aspects of an experience are remembered more vividly and accurately, the neutral details of the same experience may be less accurately recalled. This trade-off implies a focused allocation of cognitive resources toward emotionally salient information during an event, enhancing the memory of these aspects. Conversely, less attention and hence fewer cognitive resources are directed toward neutral, peripheral details, resulting in poorer recall of these elements; for instance, an eyewitness might only remember the suspect’s fierce expression, while overlooking their attire. The Fuzzy Trace Theory (FTT) explains the impact on false memories from the perspective of different components of emotional content; it suggests that, when individuals experience an event, they store two opposing memory traces (Brainerd et al., 2018). Finally, the verbatim trace stores specific details of the experience, such as remembering specific items like “apple” and “canary,” while the gist trace involves processing the meaning, such as categorizing an apple as fruit and a canary as an animal. During the recall phase, extracting the gist trace triggers false memories, while extracting the verbatim trace inhibits false memories. In terms of valence, negative emotions strengthen gist traces, leading to more false memories. Separately, regarding arousal, a moderate level of arousal enhances verbatim traces, reducing false memories, but a greater level of arousal weakens verbatim traces, increasing false memories.

Researchers have used different paradigms to investigate emotion-related false memories. The Deese–Roediger–McDermott (DRM) paradigm dominates in studies on spontaneous false memory (Deese, 1959; Roediger and McDermott, 1995). In this paradigm, participants first learn a list of associated words (e.g., “moonlight,” “bed,” “pillow,” “night”), which are semantically related to critical lure words (e.g., “sleep”) that are not presented in the list. Higher recall or recognition of the critical lure words in the subsequent test indicates a greater generation of false memories by the participant. The DRM paradigm is also suitable for studying emotion-related false memory as researchers can readily manipulate the valence of words to examine the influence of different emotions on false memory. To verify whether the results obtained from the DRM paradigm can be generalized to other materials and paradigms, researchers have also used the picture paradigm (Koutstaal and Schacter, 1997). In this paradigm, participants learn several categories of pictures derived from real life (e.g., a set of baby photos, a set of train photos) and manipulate their emotions by changing the valence and arousal levels of the pictures. A category of pictures not previously presented is used as critical lure stimuli to measure the false-memory effect. Therefore, in both the DRM paradigm and the picture paradigm, the valence and arousal levels of the memory materials can be manipulated, and differences in the manipulation of valence and arousal may lead to different results (Chang et al., 2021). In addition, the picture paradigm uses recognition tests, while the DRM paradigm combines both recognition tests and recall tests, where researchers may use a single recognition test or recall test, or they may have participants first complete a recall test and then a recognition test.

In summary, researchers have used various materials to create false memories, but the results are mixed. This prompts the question of whether there is a distinction between false memories induced by words and pictures and whether the use of different memory tests affects the outcomes. Finally, with regard to emotional variables, some previous studies did not control for either valence or arousal, while others only controlled for one or the other. Does a difference in controlling valence and arousal have an impact on research results? This review elaborates on the specific directions of the influence of emotions on false memories in studies using words and pictures as memory materials. Respectively, the aims of this investigation were to explore (1) the differences in false memories induced by picture and word materials, (2) the impact of using different tests in experiments using words as memory materials on the generation of false memories, and (3) the impact of control over valence and arousal on the results and whether these variables should be controlled in future studies.

## 2 Research on spontaneous emotional false memory

There are two ways in which emotions are generated when individuals experience events: either the events themselves carry emotions, or said individuals have already generated certain emotions before experiencing certain events. In other words, traumatic events can trigger negative emotions, and the emotions individuals feel before recalling memories of neutral events may also differ. Researchers mainly use word and picture memory materials to induce false memories, a process which is largely supported by the availability of standardized material databases, such as the IAPS. Studies employing word memory materials can be roughly divided into two categories: (1) content studies using DRM word lists with inherent emotional valence and (2) context studies that first induce emotions through other means (e.g., music, videos) and then ask participants to memorize neutral DRM word lists. Experiments that use pictures to naturally induce emotions in participants are considered content studies, while experiments that first induce a certain emotion in subjects and then have them recall neutral pictures are considered situational studies.

### 2.1 Word materials

#### 2.1.1 Content research

Budson et al. (2006) were the first to use DRM lists with negative emotional valence. In their work the unrevealed critical lure (e.g., “danger”) and the list words that participants were required to learn (e.g., “risk,” “harm,” and “threat”) were all negative in valence and semantically related. Participants were presented with negative valence lists and neutral lists of equal word length, and no significant difference in false memory between the negatively valenced and neutral lists was found on the recognition test. However, their method had a limitation: the backward associative strength (BAS) of the negative and neutral lists did not match. BAS refers to the strength of the association between list items and the critical lure. The greater the BAS value, the easier it is for participants to associate with the critical lure and the more likely a false memory will occur, while, conversely, the lower the BAS value, the less likely false memory will occur (Gallo and Roediger, 2002). To overcome this limitation, Howe (2007) controlled the BAS of the lists, and participants always completed the recall test before the recognition test. Under these conditions, the false-recall rate for the neutral valence list was greater than that of the negative valence list, and the false-recognition rate for the negative valence list was greater than that of the neutral valence list. Sharkawy et al. (2008) subsequently replicated Howe’s experiment but did not obtain consistent results: they ultimately found no difference in false recall between the two lists, but they did observe more false recognition for the negative critical lure.

Brainerd et al. (2008) offered an explanation for these contradictory findings from an alternative perspective—namely, that different studies have differed in the control of valence and arousal. To address this, they manipulated valence and arousal using affective norms for English words (Bradley and Lang, 1999) and a 9-point scale and examined the impact of different emotional valences on false memory while controlling for arousal. Subsequently, Dehon et al. (2010) conducted a similar experiment and controlled for the concreteness of the word lists. In Brainerd et al.’s study, positive emotions resulted in fewer false memories on the recognition test, while negative emotions led to more false memories. However, Dehon et al. found that, regardless of the type of test used, both negative and positive emotions increased false memory. This discrepancy in findings could be due to differences in word list concreteness, as some studies have shown that the concreteness of words in DRM lists can influence the false-memory effect (Hirshman and Arndt, 1997).

#### 2.1.2 Contextual research

Storbeck and Clore (2005) were the first to study the influence of emotions on false memory by inducing emotional states in participants through music. In their study, they first induced emotional states in participants using music with different valences and then presented the DRM list. The recognition test showed that participants in the positive emotion condition recalled more critical lures compared to those in the negative emotion condition, while participants in the negative emotion condition recalled fewer critical lures compared to those in the neutral emotion condition. In other words, positive emotions increased false memory and negative emotions decreased false memory. In subsequent experiments, Storbeck (2013) also induced emotions using music; in Experiment 1, individuals in positive and neutral emotional states produced more false memories. Meanwhile, in Experiments 2 and 3, emotions were induced using pictures selected from the IAPS, and the level of arousal was controlled for different lists. The recognition test indicated that the influence of emotions on false memory was due to valence rather than the arousal level.

Storbeck’s conclusion emphasizes the importance of valence in the influence of emotion on false memory. In contrast, some researchers believe that the impact of emotion on false memory is due to arousal. Corson and Verrier (2007) induced a series of discrete emotions using a combination of music and guided-imagery techniques, then tested the participants’ recognition memory after they memorized DRM lists. Van Damme et al. (2017) repeated Corson and Verrier’s experiment with methodological improvements and using delayed-recognition tests, free-recall tests, and immediate-recognition tests in three experiments. In addition, two control conditions were added, one with neutral emotion induction and the other with no emotion induction, to test whether different experimental manipulations would lead to different results. Contrary to Storbeck’s results, these experiments suggested that the level of arousal affected false memory rather than valence. However, these studies differ in the specific direction of the impact of arousal on false memory: Corson and Verrier found that high arousal led to more false memory than low arousal, while Van Damme et al. found that low arousal led to more false memory than high arousal.

#### 2.1.3 Summary of word material

Regardless of whether valence and arousal were controlled, DRM content studies to date have not reached consistent conclusions, and these differences may be attributable to the variable natures of the word lists used, such as the existence of differences in the BAS values of the word list and the concreteness of the words. Moreover, both recall and recognition tests have also been confounded in existing research. DRM context research has also not yielded consistent results, even when controlling for valence and arousal. It is difficult to determine whether the impact of emotion on false memory is caused by arousal or valence effect based on the existing data. The differences in experimental manipulations, such as the specific methods of emotion induction, may have contributed to the contradictory findings. Some studies have compared the effectiveness of various emotion-induction methods; for instance, Jallais and Gilet (2010) found that autobiographical recall was more effective in inducing emotions of different valences and arousal levels and, When comparing autobiographical recall to film induction methods, Salas et al. (2012) concluded that autobiographical recall was more conducive to inducing high-arousal emotions. Additionally, when comparing film induction methods to music induction methods, Van der Does (2002) discovered that music induction was more effective at evoking sadness.

### 2.2 Image materials

#### 2.2.1 Content research

Using pictures as memory materials, Choi et al. (2013) presented participants with positive images (e.g., kittens, puppies), neutral images (e.g., bookshelf, chair), and negative images (e.g., nuclear bomb, warship), each accompanied by corresponding textual labels. Subsequent recognition tests measured memories for the textual labels, and participants were observed to make fewer memory errors relating to negative items. Zheng et al. (2018) used similar picture materials to directly measure false memory for the pictures and recorded electroencephalogram data during the recognition tests. Their results were consistent with Choi and Kensinger’s findings and, additionally, event-related potential data showed that negative emotional pictures exhibited a stronger parietal old/new effect compared to neutral pictures, which is related to the retrieval process, suggesting that people are more likely to remember negative stimuli (Rugg and Curran, 2007). Unlike the images used in the previous experiments, Bookbinder and Brainerd (2017) based their study on three primary images and generated more images by altering their colors, flipping their orientation, or changing both parameters simultaneously. A subsequent recognition test used a conjoint recognition modeling based on FTT (Brainerd et al., 2022), which can distinguish the effects of verbatim retrieval and gist retrieval on false memory, thus determining which type of retrieval is influenced by valence. This recognition test revealed that negative emotions produced more false memories. In addition, the parameter-estimation data of the conjoint recognition model indicated that negative emotions enhance gist memory while impairing verbatim memory. However, as noted above, the materials used by Bookbinder and Brainerd were obtained by flipping and altering the colors of a small number of images, rendering all these images visually similar. Unlike the independent images used in other experiments, this experimental manipulation may have reminded participants of the theme of a set of images, making them more likely to generate false memories based on gist traces (Farris and Toglia, 2019).

Some studies used a series of pictures to narrate a story (a girl returning home after a trip), and manipulates the participants’ emotions by changing the outcome of the story (the girl’s home being ransacked by a robber or the girl finding a gift prepared by a stranger at home). The critical lure is the undisclosed reason for the event (the reason why the girl entered the room). Mirandola et al. (2014) were among the first to use narrative pictures as memory materials to study emotional false memories, employing materials that contained different scripts (e.g., a bicycle trip) with each script story ending differently. Half of the script stories featured negative and highly arousing content (e.g., a boy getting hit by a car, with blood around), while the other half featured neutral content with low arousal (e.g., a boy crossing the street without any accidents). Participants underwent a recognition test after viewing the script stories, and the primary result was that negative, high-arousal script content could reduce false memories. Following this, Melinder et al. (2017) used similar approaches and incorporated positive emotions into consideration. The results of the recognition test found that, compared to neutral emotions, both positive and negative emotions could reduce false memories.

#### 2.2.2 Contextual research

Mirandola and Toffalini (2016) used picture materials as memory aids, with subjects learning the picture material before entering the retrieval phase. Prior to this phase, IAPS pictures were used to induce positive, negative, and neutral emotions, respectively. Both positive and negative emotions triggered the same level of arousal, which was greater than that of neutral emotions. Following this, a recognition test was employed to investigate the impact of emotion valence and arousal on false memories. The results indicated that the groups exposed to positive and negative emotions had lower rates of false memories than the group exposed to neutral emotions, with no significant difference in false memories between the positive and negative emotion groups. There was a correlation between the valence assessed by participants and the rate of false memories, while the level of arousal assessed by participants was negatively correlated with false memories. Different from the studies mentioned above, Mirandola and Tofifalini induced emotions prior to the retrieval phase, rather than during the encoding phase, and this may have led to differences in the results. Some studies have found that stress arousal induced before the retrieval phase increases false memories (Diekelmann et al., 2011; Pardilla-Delgado et al., 2016), while other research suggests that psychological stress does not have a significant effect on processing during the retrieval phase (Smeets et al., 2008).

#### 2.2.3 Summary of image material

Studies on emotional false memories using image materials have not yielded consistent results, regardless of whether valence and arousal levels were controlled. First, some studies have employed pictures that depict a story, with intrinsic logical connections between them, and this inherent causal relationship may have influenced the occurrence of false memories. Second, in experiments using pictures without logical relationships, some investigators have used utilized images that are highly similar or even difficult to distinguish, while others have used pictures that are more easily differentiable from each other. Finally, inducing emotions during either the encoding phase or the retrieval phase could also be a reason for the observed differences in results.

## 3 Discussion on contradictory findings

The differences in the experiments mentioned in the text are displayed in Table 1. Below, we will discuss these differences in detail.

**Table 1:** Comparison of differences between experiments.

Study	Context/content	Materials	Memory test	Whether to control for valence and arousal levels?
Budson et al. (2006)	Content	Words	Recognition	No
Howe (2007)	Content	Words	Recall and recognition	No
Sharkawy et al. (2008)	Content	Words	Recall and recognition	No
Brainerd et al. (2008)	Content	Words	Recognition	Yes
Dehon et al. (2010)	Content	Words	Recall and recognition	Yes
Storbeck and Clore (2005)	Context	Words	Recognition	No
Storbeck (2013)	Context	Words	Recognition	Yes
Corson and Verrier (2007)	Context	Words	Recognition	Yes
Van Damme et al. (2017)	Context	Words	Recognition	Yes
Choi et al. (2013)	Content	Images	Recognition	Yes
Zheng et al. (2018)	Content	Images	Recognition	Yes
Bookbinder and Brainerd (2017)	Content	Images	Recognition	Yes
Mirandola et al. (2014)	Content	Images	Recognition	No
Melinder et al. (2017)	Content	Images	Recognition	No
Mirandola and Toffalini (2016)	Context	Images	Recognition	Yes

### 3.1 Different material properties

The DRM paradigm continues to dominate the study of false memory, potentially due to personal biases (Pezdek and Lam, 2007). However, while researchers may favor the DRM paradigm because of its ability to produce powerful false-memory effects, its generalizability has consistently been questioned. Picture materials may provide a useful alternative for false memory research; for example, eyewitnesses may be asked to recognize the face of a criminal suspect or a photo of the weapon used. It is also important to question whether the results obtained through the DRM paradigm can be generalized to other materials and paradigms. It has already been established that individuals display inherent differences in memory after viewing pictures and words, with picture memory typically being superior to word memory, a finding known as the picture-superiority effect. Experiments by Schacter et al. (1999) showed that the memory advantage for pictures often stems from their more distinctive encoding compared to words. Ensor et al. (2019) further elucidated the impact of differences in the physical properties of pictures and words on the false-memory effect, showing that, when words are relatively more distinctive than pictures, the memory advantage of pictures can be weakened or even reversed. In addition, research by Grady et al. (1998) has demonstrated that pictures engage memory-related regions in the brain more effectively than words do, leading to a wider range of brain activation, and this phenomenon is particularly pronounced when the stimuli are emotionally charged; in particular, highly arousing pictures activate the bilateral or right temporal lobes, while words activate the left temporal lobe. The lateral prefrontal cortex processes negative stimuli, while the medial prefrontal cortex processes positive stimuli, and the valence effect of pictures is stronger than that of words (Kensinger and Schacter, 2006). Therefore, the inherent differences between picture and word materials mean that conclusions cannot be extrapolated from experiments using different materials.

It is also difficult to measure the emotional false-memory effect of a certain material using one or even a few quantitative indicators. For false memory experiments using the DRM paradigm, the strength of the associations, specificity, and the length of the word list mentioned in the previous text all impact the false-memory effect. For false memory experiments using pictures, in addition to the similarity of the picture materials, as mentioned above, differences in the number of pictures encoded by participants may also lead to variable results. There is evidence that increasing the number of samples for each category will produce stronger gist traces (Powell et al., 1999), leading to an increase in false memory. Therefore, it can be concluded that false memory is sensitive to many different variables, and a systematic examination of the impact of these different variables is necessary.

### 3.2 Impact of memory testing

The picture paradigm for false memory typically uses recognition tests. However, in studies using the DRM paradigm, researchers may use recognition tests or recall tests in isolation, or they may have participants engage in recall tests before recognition tests. Using different tests or a combination of tests can simulate situations in which false memories occur in real life. For example, police may request that eyewitnesses recall or identify the appearance of a suspect, or they may first have eyewitnesses recall the crime scene before conducting recognition tests. However, both recall and recognition tests yielded inconsistent results in the experiments performed by Howe (2007) and Sharkawy et al. (2008), as described earlier. Recall tasks require participants to search for specific information, while recognition tasks provide more specific cues. Evidence suggests that recognition tests are more likely to produce false memories, while recall tests have the opposite effect (Seamon et al., 2003). In cases where a combination of tests is used, prior recall tests can influence subsequent recognition tests (Roediger et al., 2001), and, when participants can recall all the words in a word list, false recognition decreases (Gallo, 2004). Recognition tests are more sensitive to items with emotions and are more likely to produce false memories for emotional material (Brainerd et al., 2014). In a recent study, researchers controlled for the valence and arousal levels of 32 DRM lists, having subjects complete recall and recognition tests. The results showed that there was an interaction between valence and arousal in the recall test, but this interaction was not observed in the recognition test (Chang et al., 2021). In conclusion, the aforementioned studies show that different memory tests have different effects on false memory, and the order and combination of tests used can also affect subsequent recognition tests. However, the exact reasons for these effects require further research.

### 3.3 Control of valence and arousal levels

In the analysis of this article, the control of valence and arousal cannot resolve the contradictions in the research on emotional false memories. Different from our viewpoint, Bookbinder and Brainerd (2016) believe that controlling the valence and arousal of memory content could help resolve the contradictions currently present in false memory research. They explain the contradictory findings on emotional false memory as context–content conflicts. They believe that, in context research, positive valence increases, while negative valence decreases false memory under controlled valence and arousal levels. In content research, negative valence increases, and positive valence decreases false memory. However, even Bookbinder and Brainerd’s own research fails to support this view (Brainerd et al., 2008; Bookbinder and Brainerd, 2017). In both content studies, the arousal level of the encoded materials was controlled, but the former found that positive emotions reduce false memory and the latter found that positive emotions increase false memory. The authors suggest that this may be because verbatim memory is more sensitive to different materials. Studies consistent with this view indicate that the increase in positive emotions is more dependent on the controlled fine processing of the left amygdala and prefrontal cortex, which are more likely to be influenced by different experimental manipulations (Pessoa, 2018). Whether to control for valence and arousal is the topic explored in this article. In subsequent discussion, we will elaborate on the issues that controlling valence and arousal brings, as well as why it’s necessary to go beyond the dimensions of valence and arousal to examine the impact of discrete emotions on false memories.

## 4 Summary and outlook

The impact of emotions on false memory has long been a concern in the fields of law and medicine. Extensive studies have shown that emotions can affect false memory, but there is no consistent explanation of how this occurs. This article reviewed the literature and summarized the results and methodological issues of previous studies.

Studies using words as memory materials have enhanced people’s understanding of the constructive nature of memory and the susceptibility to memory errors. However, the results obtained from word lists are not consistent with situations using other materials. Researchers appear to reach the same conclusions using different materials, which is not consistent with the actual situation. Future research must clarify the inherent differences in inducing false memory using different materials such as words, pictures, videos, music, and so on. In addition, Whittlesea et al. (2005) raised doubts about the DRM paradigm from a unique angle. They embraced the DRM paradigm and found that participants’ false memories were caused by surprise induced by the critical lure (note, the critical lure was more recapitulative and connected to other learned words). Further studies revealed that, when participants consciously suppressed their surprise at the critical lure, the DRM effect vanished. Interestingly, one study suggests that surprise can elicit negative emotions (Topolinski and Strack, 2015), and whether surprise is truly an emotion remains a controversial question. Some have indicated that surprise does not always have a certain level of valence, which is a characteristic of each emotion (Gerten and Topolinski, 2019; Ortony, 2022). Therefore, it is evident that this issue is quite complex, and it is crucial to explore the impact of surprise caused by the critical lure on false memories in future research. On the other hand, the false memory induced by word lists is different from the false memory of real events in judicial and psychological therapy contexts (DePrince and Freyd, 2004), as each group of words or pictures is relatively independent and lacks logical relationships. The method mentioned earlier involves using a collection of pictures that depict a story as memory materials, which, to some extent, addresses the problem of the lack of logical relationships between learning items in traditional paradigms.

Emotion-induced false memories are sensitive to many different variables, and even subtle changes in the experimental design can lead to changes in the direction of emotion-induced memory errors. Additionally, recall and recognition tests, respectively, have different effects on memory errors, but they are often confused with each other in the existing literature. A recent study (Wiechert et al., 2024) used meta-analysis and replication research methods to uncover that negative valence does not systematically affect false memory; instead, the formation of false memories depends upon how false memories are tested; in the recall test, valence had no effect on false memory, while, in the recognition test, the effect of false memory may be attributed to response bias. Additionally, Yüvrük and Kapucu (2022) found that the effect of valence was non-significant when recognition responses were controlled for response bias. These results indicate that future research should quantify the impact and specific contributions of different manipulations on memory errors, and examining the impact of emotions on false memory from the perspective of valence and arousal alone is far from sufficient. Among previous studies, only some controlled for valence and arousal, and the conflation of the two could have caused variability in the results. Should valence and arousal be controlled? While the valence and arousal of emotions might differently influence false memories, it’s not sufficient to limit research solely to the effect of emotional arousal on memory (Levine and Pizarro, 2004). Individuals may experience specific emotions like happiness, fear, despair, or anger, but they are never merely “aroused.” Previous studies have shown that emotional experiences cannot be fully captured by just two dimensions (Panksepp, 1992; Barrett, 1998). For example, the circumplex model of emotion struggles to accurately depict rare but complex emotions like shame, guilt, or jealousy, and these emotions are common in the real situations where false memories are created. Thus, confining research to just valence and arousal essentially lacks external validity. Specific emotions have adaptive functions, enabling us to respond appropriately to environmental changes (Howe, 2011); for instance, fear and anger are similar in valence and arousal, but fear tends to make individuals avoid threats, whereas anger inclines individuals toward eliminating threats (Cunningham and Brosch, 2012). In the field of false memory, there are also numerous examples that support this view. A study on the emotion congruence of discrete emotions (Bland et al., 2016) induced participants’ fear and anger through film clips (with no significant difference in valence and arousal) and asked participants to recall DRM lists with fearful and angry themes. In the subsequent recognition test, participants erroneously remembered critical lures consistent with their emotional state. In another study on discrete emotions, (Van Damme et al., 2017) conducted an experiment in which participants were induced to experience corresponding emotions by having them empathize with the content of the slides. The results from the recognition tests showed that participants under hope and fear conditions produced more false memories than did those under happy or despair conditions. Furthermore, studies on clinical populations have evidence that individuals with PTSD, depression, and a history of trauma are more prone to false memories related to their psychological disorders (e.g., trauma-related stimuli), regardless of the valence and arousal (Otgaar et al., 2017). The above results cannot be explained solely by the dimensions of arousal and valence, indicating that limiting research to investigating only valence and arousal does not facilitate a clear understanding of the relationship between emotions and false memories.

Contrary to what previous researchers have advocated for in controlling valence and arousal levels, this paper innovatively proposes that we should go beyond the dimensions of valence and arousal to explore the impact of emotions on false memories. It is worth mentioning that this paper focuses on spontaneous emotional false memories, and similar results have also been demonstrated in studies of implanted false memories. In their work, Sharma et al. (2023) reviewed 39 studies to explore the relationship between emotions and implanted false memories and found that the impact of emotions on implanted false memories depends upon the type or aspect of emotional measurement; specifically, the valence of emotions did not affect the generation of false memories, and when information was recalled with a delay, the arousal of emotions also did not have an impact on false memory. Moreover, stress and short-term distress experienced by subjects before encoding reduced the implanted false memories produced by the subjects, while prolonged distress, anger, and greater stress increased the implanted false memories produced by the subjects. This outcome might be because the two dimensions of the circumplex model of emotions cannot explain the dynamic nature of emotional changes. Emotional experiences are often rapidly changing, comprised of both continuous and momentary variations (Scherer, 2005).

Although it may be convenient to describe emotions simply in terms of valence and arousal, two emotions with the same valence and arousal (such as fear and anger) can have different effects on false memory. Moreover, limiting research to valence and arousal neglects the adaptive nature of emotions. Therefore, future research should not only control for valence and arousal dimensions but also explore the false memories induced by specific emotions.

## Author contributions

HY: Writing – original draft. YZ: Writing – review & editing. ZL: Writing – review & editing.
